# A Rietveld refinement method for angular- and wavelength-dispersive neutron time-of-flight powder diffraction data

**DOI:** 10.1107/S1600576715016520

**Published:** 2015-10-13

**Authors:** Philipp Jacobs, Andreas Houben, Werner Schweika, Andrei L. Tchougréeff, Richard Dronskowski

**Affiliations:** a Chair of Solid-State and Quantum Chemistry, Institute of Inorganic Chemistry, RWTH Aachen University, Aachen, D-52056, Germany; b European Spallation Source ESS, Lund, SE-22100, Sweden; c Jülich Center for Neutron Science and Peter Grünberg Institute PGI, Forschungszentrum Jülich, Jülich, D-52425, Germany; d Department of Chemistry, and Poncelet Laboratory of Mathematics in Interaction with Physics and Informatics, Lomonosov Moscow State University, Independent University of Moscow, Moscow Center for Continuous Mathematical Education, Bolshoi Vlasevsky Per. 11, Moscow, 119002, Russian Federation

**Keywords:** Rietveld refinement, neutron diffraction, angular-dispersive, wavelength-dispersive, powder methods, *VITESS*, POWTEX, POWGEN

## Abstract

This paper introduces a two-dimensional extension of the well established Rietveld refinement method for modeling neutron time-of-flight powder diffraction data. The novel approach takes into account the variation of two parameters, diffraction angle 2θ and wavelength λ, to optimally adapt to the varying resolution function in diffraction experiments. By doing so, the refinement against angular- and wavelength-dispersive data gets rid of common data-reduction steps and also avoids the loss of high-resolution information typically introduced by integration. In a case study using a numerically simulated diffraction pattern of Rh_0.81_Fe_3.19_N taking into account the layout of the future POWTEX instrument, the profile function as parameterized in 2θ and λ is extracted. As a proof-of-concept, the resulting instrument parameterization is then utilized to perform a typical refinement of the angular- and wavelength-dispersive diffraction pattern of CuNCN, yielding excellent residuals within feasible computational efforts. Another proof-of-concept is carried out by applying the same approach to a real neutron diffraction data set of CuNCN obtained from the POWGEN instrument at the Spallation Neutron Source in Oak Ridge. The paper highlights the general importance of the novel approach for data analysis at neutron time-of-flight diffractometers and its possible inclusion within existing Rietveld software packages.

## Abstract 2

This paper introduces a novel approach to Rietveld refinements of two-dimensional angular- and wavelength-dispersive powder diffraction data as measured at time-of-flight neutron diffraction instruments. To do so, the authors’ *ansatz* for diffraction data obtained from the POWGEN diffractometer has been verified, and furthermore its feasibility and benefit in simulations for the novel POWTEX instrument presently under construction are demonstrated.

## 1. Introduction   

Since the early days of the ingenious Rietveld method (Rietveld, 1969 ▸) in the 1960s, the method has become widely applied because it allows crystallographic and, using neutron data, even magnetic structure investigations on powdered, polycrystalline samples. The slowly but steadily evolving computational power in the 1970s and the proper description of the individual peak shapes were two essential steps for moving away from the simple interpretation of total integrated intensities towards a least-square fit of the full diffraction profile. This holds especially true for overlapping peaks as encountered in low-symmetry structures or in multiphase samples. As a presumably lucky coincidence, the neutron powder diffractometer used by Rietveld appeared to deliver Gaussian-like shapes for the Bragg peaks, just like in the case of modern monochromatic neutron diffractometers. Even though the peak shapes of time-of-flight (TOF) data are certainly more complicated (Von Dreele *et al.*, 1982 ▸), the Rietveld method was soon applied for TOF instruments as well.

When trying to properly describe the peak shape in general, one immediately realizes that a plethora of different effects contribute to the way it looks. The first aim is to separate the instrumental and geometrical effects, *e.g.* the asymmetry caused by the umbrella effect (Finger *et al.*, 1994 ▸; van Laar & Yelon, 1984 ▸) or the change of the peak widths (FWHM, full width at half-maximum) with the scattering variable by changing instrumental resolution, from the intrinsically more interesting sample effects (*e.g.* crystal structure, particle size, microstrain or texture effects). In current Rietveld algorithms, there are numerous approaches to model the influences of all such effects on the peak shape and intensities (Avdeev *et al.*, 2007 ▸; Dollase, 1986 ▸; Lutterotti *et al.*, 1999 ▸; March, 1932 ▸; Popa, 1998 ▸; Rodriguez-Carvajal, 1997 ▸; Stephens, 1999 ▸). One example is the incorporation of classical texture analysis into the Rietveld method as done by the *MAUD* software (Lutterotti *et al.*, 1999 ▸; http://maud.radiographema.com/). Here, the intensity is described as a function of the scattering angle 2θ and the polar angle φ along each Debye–Scherrer cone. Hence, the data acquisition has to account for both variables, which is normally performed using a (one-dimensional) position-sensitive detector to save measurement time or, even better, by using two-dimensional detectors which, in addition, reveal potentially sharp textures.

Modern powder diffractometers (Chapon *et al.*, 2011 ▸; Huq *et al.*, 2011 ▸; Kamiyama *et al.*, 1995 ▸; Peters *et al.*, 2006 ▸) at advanced neutron spallation sources (Fischer, 1997 ▸; Ikeda, 2005 ▸; Lengeler, 1998 ▸; Lisowski & Schoenberg, 2006 ▸; Mason *et al.*, 2006 ▸) typically operate in TOF mode and use large area detectors, thereby generating angular- and wavelength-dispersive data. This is in contrast to classical monochromatic instruments (Fischer *et al.*, 2000 ▸; Garlea *et al.*, 2010 ▸; Hansen *et al.*, 2008 ▸; Hoelzel *et al.*, 2012 ▸; Liss *et al.*, 2006 ▸; Többens *et al.*, 2001 ▸) at continuous reactor sources as well as typical X-ray powder diffractometers. Current approaches at existing instruments therefore first reduce, transform and integrate the accumulated data to obtain the well known one-dimensional diffraction patterns (*MANTID*; http://www.mantidproject.org; Schäfer *et al.*, 1992 ▸) (*e.g.* intensity as a function of 2θ or TOF) that can be routinely treated using the standard software packages (Bruker, 2005 ▸; Larson & Von Dreele, 1994 ▸; Lutterotti *et al.*, 1999 ▸; Petříček *et al.*, 2006 ▸; Rodríguez-Carvajal, 1993 ▸, 1997 ▸). For example, the inevitable φ dependence encountered at two-dimensional detectors is integrated for each reflection to allow for standard Rietveld refinements. This is normally done by straightening the measured Debye–Scherrer cones detected as circles on flat area detectors (Elf *et al.*, 2002 ▸). Although this simple procedure has the advantage of refining diffraction data that are relatively small in size and leads to quick calculations, a significant amount of the available information is lost and cannot be exploited.

In a sense, the present situation resembles the challenges Hugo Rietveld had to meet back in the 1960s: a lack of both computing power and powerful algorithms resulted in an unsatisfying data representation. Indeed, this very data-massaging problem is known to the entire community, and it has also been said: ‘One day in the not too far distant future one may leave it curved and introduce the necessary peak shape/resolution functions into a two-dimensional Rietveld refinement’ (Kuhs & Klein, 2008 ▸).

Here we shall introduce a novel data-treatment approach for angular- and wavelength-dispersive data sets based on simulation results for the evolving TOF powder diffractometer POWTEX (Conrad *et al.*, 2008 ▸; Houben *et al.*, 2012 ▸). A few sentences covering the design of that instrument seem in order.

The POWTEX instrument will feature a four-dimensional large area detector (Modzel *et al.*, 2014 ▸) covering about 9 steradian of solid angle, and the detection of neutron events will be position sensitive (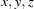) and time resolved (*t*), hence four-dimensional. The measured quantities can be readily converted to the Bragg angle θ, the polar angle φ, the detection depth *z* and the wavelength λ. For the present analysis, we will reduce the data by integrating over the polar angle φ and the detection depth *z* so that only the variables 2θ and λ remain. We will lay out all necessary steps, especially how the peak shape is parameterized using both variables and how the varying resolution function is expressed by a suitable parameterization.

Since the Rietveld method represents a least-squares fit that is totally independent of the data dimension, we want to emphasize, however, that one day in the not too distant future one may even apply a Rietveld model refinement to three-dimensional and four-dimensional powder diffraction data.

## 2. Simulations   

Because of the fact that the POWTEX instrument is currently under construction on beamline SR5a at the FRM II neutron source, real experimental data sets are not available yet. Nonetheless, it is mandatory to (approximately) know the POWTEX data ahead of the instrument’s realization, for obvious reasons; this may be accomplished as follows: instrument simulation programs using Monte Carlo methods (Lefmann & Nielsen, 1999 ▸; Lieutenant *et al.*, 2014 ▸; Wechsler *et al.*, 2000 ▸; Zsigmond *et al.*, 2006 ▸) may be used in order to obtain data sets that closely resemble those that will be obtained by the instrument in the future. Consequently, the *VITESS* program package (Lieutenant *et al.*, 2014 ▸; Wechsler *et al.*, 2000 ▸; Zsigmond *et al.*, 2006 ▸) was utilized to simulate data sets based on instrumental parameters of the POWTEX diffractometer. The main parameters included in the simulation are summarized in Table 1 ▸. For simulating the neutron source, a wavelength distribution according to the input file (‘Frm-II_thermal.dat’) of the newest *VITESS* version for SR5 was used. With respect to the resolution of the real instrument, the simulations account for the neutron-guide definition with its divergence properties, the four-disc chopper system including the double-disc pulse chopper, the sample and the detector geometry. The neutron-guide system was simulated as a polygonal approximation of the (partially) truly curved guide geometry, and it includes the design values of the reflective coating scheme (Houben *et al.*, 2012 ▸). The time resolution (Δ*t* ≃ 10 µs) is essentially defined by the double-disc pulse chopper.

The sample module determines the scattering of the neutron beam by simply generating a random location along the neutron trajectory through the sample at which the neutron is scattered out of the sample. The scattering process itself is based on structural models given as an input file to the sample module; absorption effects were neglected in these first simulations. The detector system is implemented in monitor mode, meaning that the position of each neutron count is pinpointed to the intersection of the trajectory with the detector surface. For simplicity, the detector’s spatial resolution was assumed to be perfect, since the POWTEX’s detector characteristics have not yet been defined in all details. The simulations were based on the structural models of Rh_0.81_Fe_3.19_N and CuNCN (Houben *et al.*, 2005 ▸, 2009 ▸; Liu *et al.*, 2005 ▸; Jacobs *et al.*, 2013 ▸) (see Table 2 ▸) using a large number of trajectories (1.5 × 10^12^).

After recording all neutron trajectories, the resulting data (position in 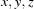; time; trajectory probability, *i.e.* intensity) were converted to three-variable data sets (2θ, λ, trajectory probability) by applying simple geometrical relations and relating TOF to wavelength. Therefore, a new two-dimensional module called ‘eval_elast2’ was implemented in *VITESS*. Herein, the resulting data were integrated over the polar angle φ and binned in 2θ and λ using bin sizes of 0.1° and 0.001 Å (corresponding to approximately a third of the time resolution). The binning mimics a possible sampling grid and is finer than the instrument resolution. Incorporating more instrumental details will be a future task which will not affect the principle of the approach presented.

## 3. Neutron powder diffraction   

As a ‘real-world’ alternative, additional neutron powder diffraction data of CuNCN were obtained at the POWGEN instrument [Spallation Neutron Source (SNS), Oak Ridge]. The measurement time was approximately 7.5 h. In addition, the background and the vanadium measurements were carried out as well. The Nexus event data of the sample, background and vanadium measurements (stored in the corresponding ‘event.nxs’ file) were treated according to the standard ‘*SNSPowderReduction*’ Python script included in the *MantidPlot* program package (*MANTID*). Nonetheless, we omitted the cylindrical absorption correction for the vanadium data and the final diffraction focusing step. Hence, in contrast to the standard *d* binning used in *MantidPlot* (logarithmic binning), in our case the event data are binned in 2θ as well as in λ using bin sizes of 0.1° and 0.001 Å, respectively, matching those for the simulated data on purpose. Subsequently the sample pattern was corrected for background and finally calibrated by the vanadium pattern (from which the vanadium reflections had already been stripped off) to account for detector efficiency and to remove the wavelength-dependent intensity distribution.

## 4. Data-analysis approach   

For all calculations presented in this work we used the MATLAB program package (http://www.mathworks.com). All refinements were carried out using functions and code explicitly written (but not yet fully optimized) for the work presented herein.

**Figure 1:**
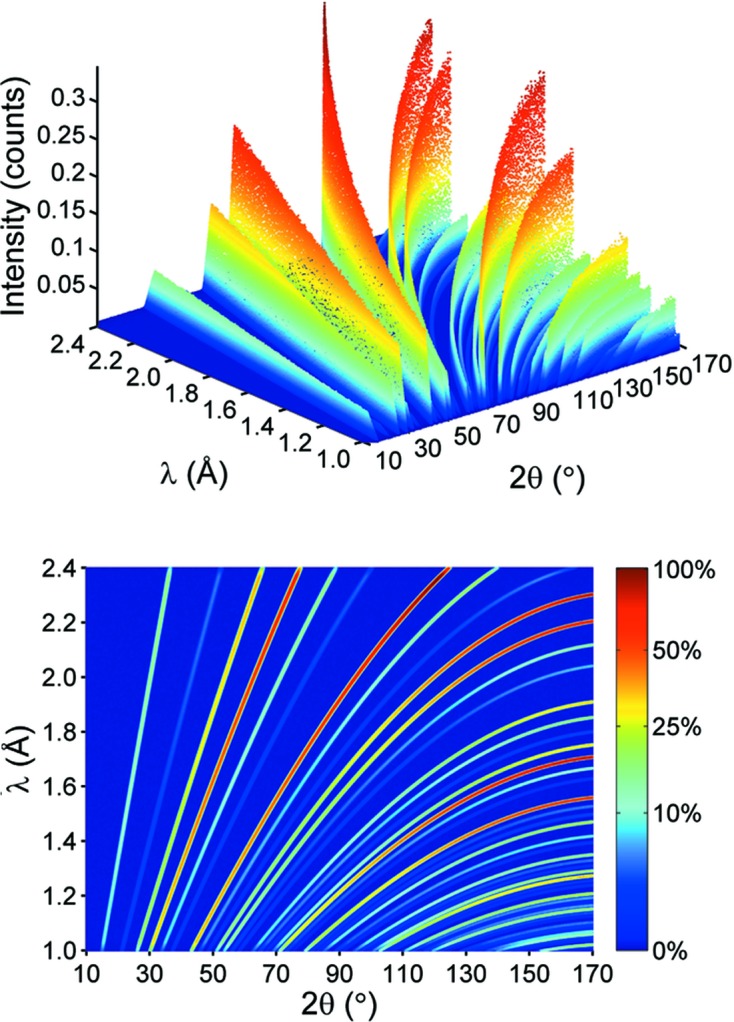
Simulated diffraction pattern *I*(2θ, λ) of Rh_0.81_Fe_3.19_N using a two-dimensional (top) and quasi-two-dimensional (bottom) representation.

We reiterate that the simulated data as stored in the output file of the *VITESS* simulation are intensities as a function of 2θ and λ. A simulated pattern for Rh_0.81_Fe_3.19_N is shown in Fig. 1 ▸. Although similar patterns are obtainable from other existing TOF instruments with large area detectors, at least in principle, almost no references to two-dimensional diffraction patterns can be found in the literature. Schäfer *et al.* (1992 ▸) provide such a plot but for further analysis the data *I*(*d*) were grouped into equally spaced *d* intervals. This simplification is typical for current approaches at TOF instruments and allows for the use of standard refinement packages.

In Fig. 1 ▸, single reflections of constant *d* spacing are now represented by *sinusoidal curves*, as given by Bragg’s law (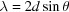) (Bragg & Bragg, 1913 ▸). Note that the width of each reflection varies with scattering angle and wavelength. In order to perform a Rietveld refinement, *i.e.* a least-squares-type analysis, one needs to find an analytical description of the diffraction pattern that is able to fit all variable parameters to the measured data set. In the two-dimensional case the calculated intensity *I*_calc_(2θ, λ) for a single-phase diffraction pattern can be expressed for every data point by
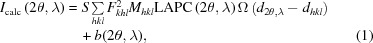
with *S* being a scaling factor, *M_hkl_* the multiplicity, 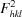 the structure factor, LAPC(2θ, λ) introducing geometrical and physical corrections, Ω(*d*_2θ,λ_ − *d_hkl_*) being the profile function that models both instrumental and sample effects, and *b*(2θ, λ) the background. The summation is done over all *hkl* reflections for each data point. LAPC comprises the Lorentz factor, absorption, preferred orientation and special corrections to the intensity distribution. As the simulation does not include absorption and preferred orientation, these and other such special effects were neglected. The Lorentz factor, on the other hand, has been accounted for and found to be proportional to *d* ^4^ for the simulated data (Von Dreele *et al.*, 1982 ▸).

The next step involves finding a suitable description of the profile function for the two-dimensional case. For one-dimensional data a lot of effort has been invested over several decades to find good profile functions describing the form and width of the reflection peaks for different instruments and neutron sources (Bacon & Thewlis, 1949 ▸; Cheetham & Taylor, 1977 ▸; Dinnebier & Billinge, 2008 ▸; Young & Wiles, 1982 ▸). A common trait of all current profile functions is that they depend on one variable only (in most cases either 2θ, TOF or *d*). In obvious contrast, analyzing two-dimensional data sets will require the reflection profile to be a function of two variables, here wavelength and scattering angle, while the rest of the parameters presented in equation (1) will essentially remain untouched.

**Figure 2:**
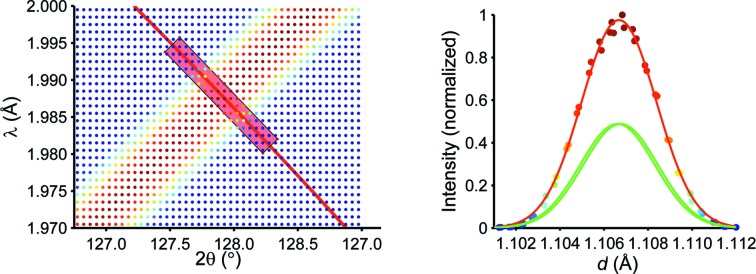
Data points contributing to a single slice indicated by the pale red rectangle (left) for which the orthogonal cut (red line) is taken at 2θ = 127.8° and λ = 1.988 Å, corresponding to a Bragg peak at *d* = 1.1067 Å. Intensity plotted against *d* value of each data point (right) in which the red line is a fitted curve using the sum of two Gaussians and the green curves represent the single Gaussians.

For the two-dimensional description of the simulated POWTEX data, an appropriate profile function Ω needs to be found. Some of the resolution effects that are relevant for the real instrument and contribute to the profile shape are accounted for in the simulations already. The elliptic neutron guide mainly determines the shape of the divergence distribution, which is of particular importance for the profile function Ω, and can be described by a sum of two Gaussians of equal height but shifted by ±δ_2θ,λ_ from the central *d_hkl_* value, resulting in a smooth symmetric beam profile. For an arbitrarily chosen data point in the diffraction pattern with 2θ = 127.8° and λ = 1.988 Å, the peak profile 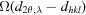 is shown in Fig. 2 ▸. One could easily imagine that the use of different amplitudes, σ and δ values for each of the single Gaussians would even allow one to approximate asymmetric peak shapes; for further information about this topic the reader is referred to Howard (1982 ▸). It might indeed become necessary to use a slightly different function for the actually measured POWTEX data but the mathematical procedure described in the following would be very similar.

Note that the profile Ω, the width σ and separation δ of the two Gaussians vary with the diffraction angle 2θ and the wavelength λ:
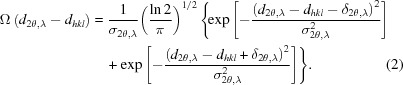
The parameters σ and δ will depend on how the intersection of the profile function with the Bragg diffraction lines is defined, namely where 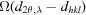 is normalized to
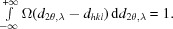
For a common treatment of all Bragg reflections, we consider Ω along curves intersecting the Bragg lines λ = 2*d*sinθ orthogonally for all values of *d*. These curves are obtained by solving the differential equation
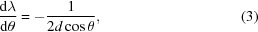
where λ and *d* have to be understood as dimensionless values. After expressing *d* through λ and θ according to the Bragg relation, we arrive at the expression
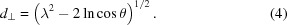
Here 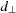 (= λ at θ = 0) is an alternative coordinate that – together with *d* – gives a new orthogonal coordinate system.

**Figure 3:**
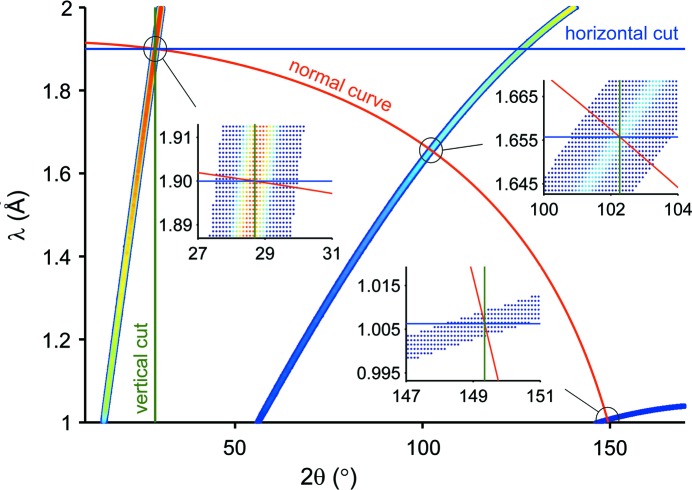
Course of the 100, 222 and 552 reflections and the resulting data of the *VITESS* simulation, together with lines for the horizontal, vertical and normal cuts. Insets show an enlarged version of the cross sections.

It is obvious that considering orthogonal trajectories to the Bragg reflections is the most appropriate description for defining the profile function Ω, and it exploits the two-dimensional information most efficiently. This is illustrated in Fig. 3 ▸ where, taking the 100, 222 and 552 reflections of Rh_0.81_Fe_3.19_N as examples, the horizontal and vertical cross sections are shown for comparison. Indeed, the horizontal cut corresponds to a monochromatic measurement and the vertical cut to a TOF measurement at a fixed angle. Additionally the inclination of the cuts orthogonal to the reflection changes over the diffraction pattern. For the 100 reflection the orthogonal cut is almost comparable to the horizontal cut, while for the 552 reflection we almost have a vertical cut. For the 222 reflection the orthogonal cut lies in between.

**Figure 4:**
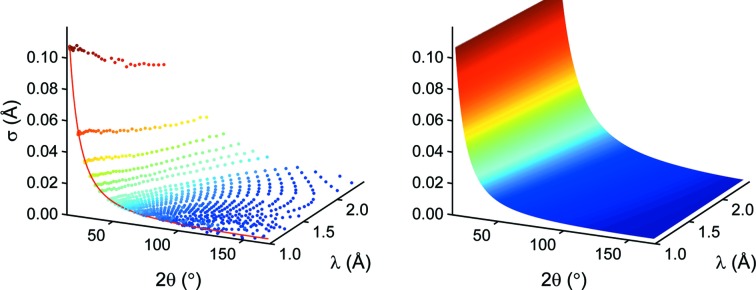
σ values extracted at a large number of different points in the diffraction pattern (left) and fitted surface to the σ values (right) with *R* ^2^ = 0.993. The red line connects data points of constant wavelength.

It is preferable to separate the instrumental contribution to the peak shape and width from additional sample effects such as strain, stress or size effects. In a real experiment, one would use standard samples to determine such parameters. In the present modeling study we will simply use our simulated and idealized sample. Extracting the σ and δ values at various points of the diffraction pattern leads to the distributions shown in Fig. 4 ▸. Similar to the Caglioti formula (Caglioti *et al.*, 1958 ▸) we find an appropriate analytical description of the instrument characteristics using the parameters σ and δ as a function of 2θ and λ:
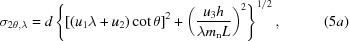


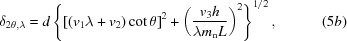
with *u*_1_ and *u*_2_ describing a wavelength-dependent angular resolution (Δθ), and *u*_3_ essentially representing the time resolution (Δ*t*) of the resolution function. The parameters *v*_1_, *v*_2_ and *v*_3_ are coefficients of the displacement function, and *L* is the distance from the chopper to the detector at position 2θ Furthermore, *h* and *m*_n_ represent Planck’s constant and the neutron mass, respectively. The refinement results of the profile parameters were obtained from a sufficiently large number of orthogonal cuts. The variations of σ and δ *versus* 2θ are similar, and both are smooth functions, as expected. It seems that there are minor deviations around 2θ = 45 and 135° coinciding with the corners of the cylindrical detector, thereby corresponding to a discontinuity in the derivative of Ω. It is straightforward, however, to define Ω in a piece-wise fashion and also separately for the three detector elements. This has not been done here, because we consider these effects as negligible at the current stage. The fit of the parameters *u*_1–3_ and *v*_1–3_ nicely matches the measured σ and δ values and yields an overall *R* ^2^ value of 0.993 (see Fig. 4 ▸).

**Figure 5:**
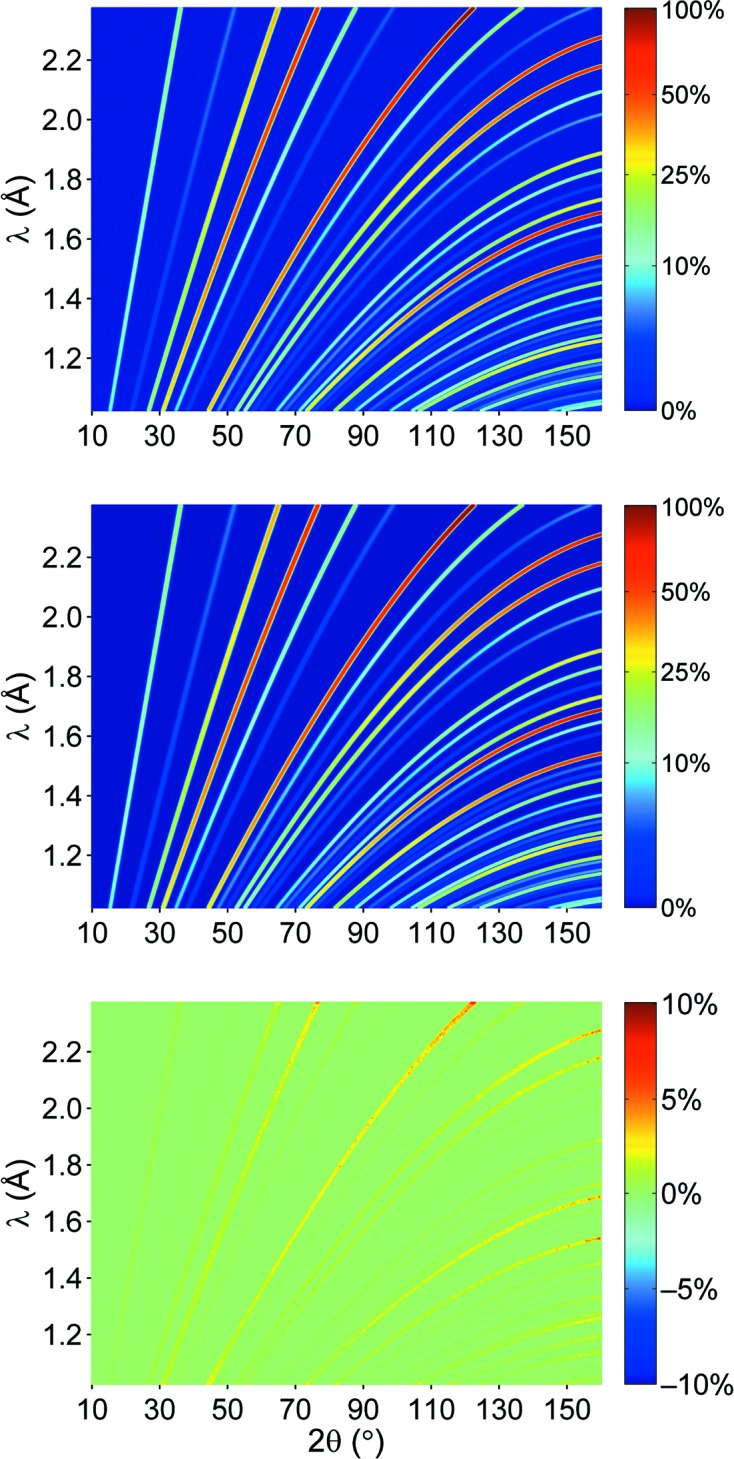
Simulated (top), calculated (middle) and differential (bottom) diffraction pattern of Rh_0.81_Fe_3.19_N at POWTEX. The color bar of each picture denotes the intensity as a percentage of the largest intensity peak in the simulated diffraction pattern.

When replacing σ and δ in equation (2) with their corresponding analytical functions, one may fit the measured data using the resulting profile function (which now depends on 2θ *and* λ) in equation (1), just like in typical ‘structural’ Rietveld refinements carried out up to the present day. The background *b* as originating from the incoherent scattering is accounted for by a single parameter. In a first attempt at structural refinement, only a limited number of parameters were refined, namely the scale *S* and background *b* as well as the lattice parameters, while keeping the internal structural parameters (atomic site, displacement parameter *etc*.) fixed. The results of the pattern fitting for the Rh_0.81_Fe_3.19_N and CuNCN phases are summarized in Table 3 ▸. Fig. 5 ▸ presents the simulated diffraction pattern, the calculated diffraction pattern and their difference (*I*_calculated_ − *I*_simulated_). The overall agreement seems to be satisfactory. Nonetheless, small deviations of the calculated pattern from the simulated data, especially at high 2θ angles and high λ values, can be observed. At high λ values the intensity in the simulated diffraction patterns falls off near the maximum value of 2.4 Å, while in the calculated pattern the intensity remains unchanged. One may note that the rather sharp diffraction lines in backscattering were imperfectly described by the too coarse binning. Of course, a finer binning is always possible for an appropriate refinement of the analytical profile description, and with the choice to limit additional computational effort to the relevant backscattering region.

**Figure 6:**
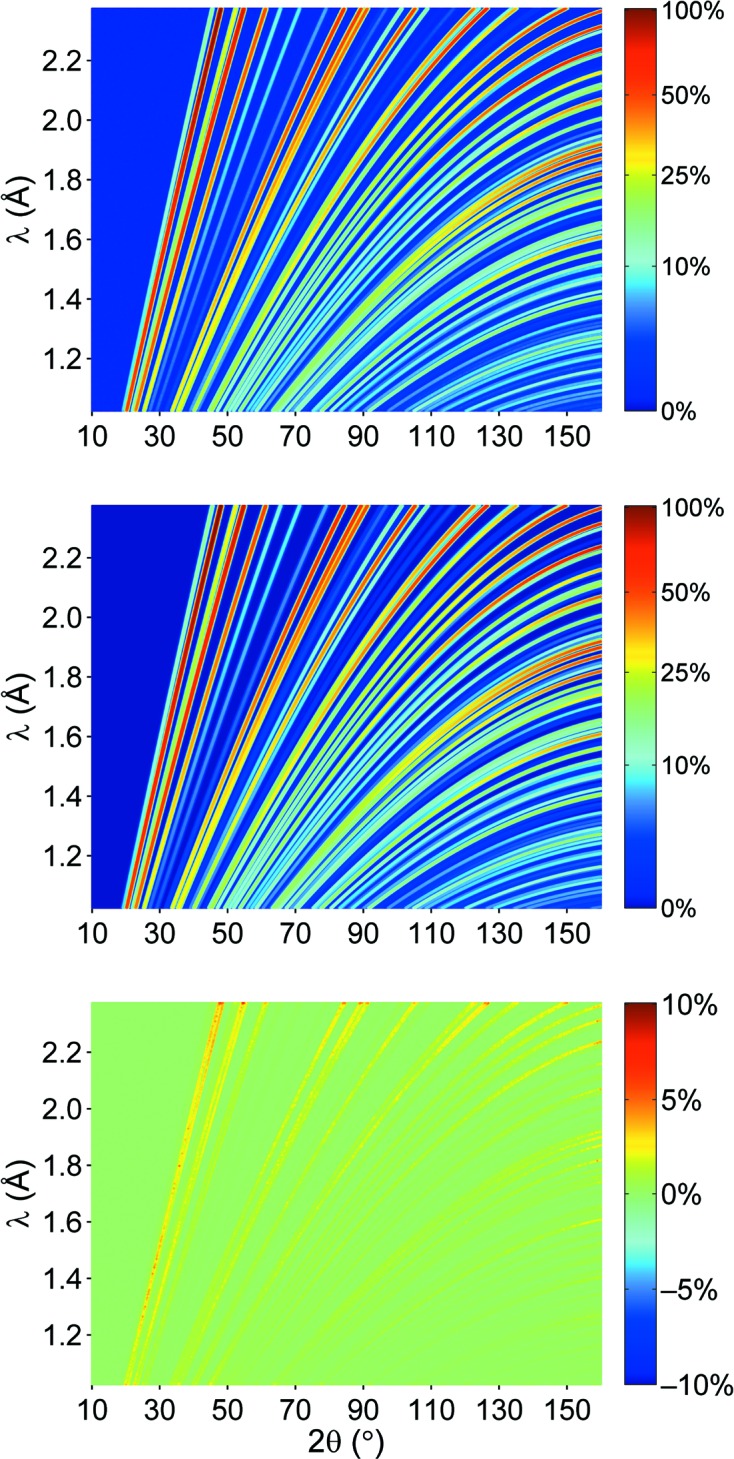
Same as in Fig. 5 ▸ but for CuNCN.

By neglecting any sample effects aside from the scattering process, the obtained parameterized profile function as resulting from the Rh_0.81_Fe_3.19_N simulation should be regarded as the *instrumental* profile function, which does not depend on the actual sample. Therefore, the profile function is the valid base for fitting the diffraction patterns of standard samples without discernible size/strain contributions. This is exemplified by the Rietveld refinement of simulated CuNCN data but using the *identical* parameterization of the profile function as in the Rh_0.81_Fe_3.19_N case. The diffraction pattern is shown in Fig. 6 ▸. Note that even regions with severe peak overlap are very well described, and the calculated intensities nicely match the simulated ones over almost the entire diffraction pattern. The results of the pattern fitting are summarized in Table 3 ▸. Minor deviations are only observed at large diffraction angles and wavelength, as mentioned above, but otherwise the agreement is very good.

As noted by an insightful reviewer, the aforementioned strategy – refining against a *simulated*, Monte Carlo derived data set by a novel two-dimensional Rietveld method and gauging the quality of the latter *only* by comparing simulated and theoretical intensities – might look questionable because experimental data are totally lacking; we reiterate, however, that there are no experimental data since the POWTEX machine is still under construction. Nonetheless, it is possible to experimentally test the novel method although real data must then come from a different source. To do so and follow the strategies laid out in the preceding part, we have tested our approach using a *real* data set for CuNCN which was obtained at the POWGEN instrument. Because of the natural wavelength dependence of the moderator pulse and the heart-shaped detector arrangement of POWGEN, the instrument yields almost constant resolution 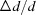, while a varying resolution function is more appropriate for the required use of pulse-shaping choppers at the POWTEX instrument.

**Figure 7:**
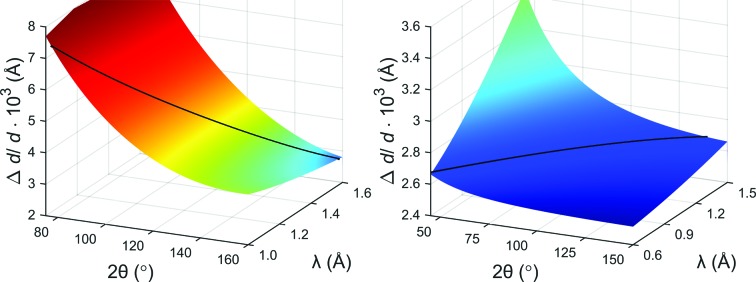
Comparison of resolution functions for POWTEX (left) and POWGEN (right). The black curve is a visualization of points belonging to a single reflection with *d* = 0.8 Å.

The flexibility of our new approach which includes a varying resolution function also has an important benefit for the instrument design, simply because the detector shape is much simpler and more economical in terms of construction costs, *i.e.* cylindrical and covering a large solid angle. The profile function is mainly determined by the FWHM of each reflection independent of the actual 2θ and λ value of the data point. This is depicted in Fig. 7 ▸, where the resolution function for the POWTEX instrument (left) is compared with that of the POWGEN instrument (right). For an arbitrarily chosen reflection at *d* = 0.8 Å (black line) it is obvious that POWTEX’s resolution changes with 2θ and λ while for the POWGEN instrument it essentially remains the same. In particular for the CuNCN measurement, we note that the sample exhibits microstrain discernible by the FWHM of each reflection, an effect which had to be corrected by applying a strain correction (DST^2^) in the quartic form for Laue class *mmm* (Stephens, 1999 ▸).

**Figure 8:**
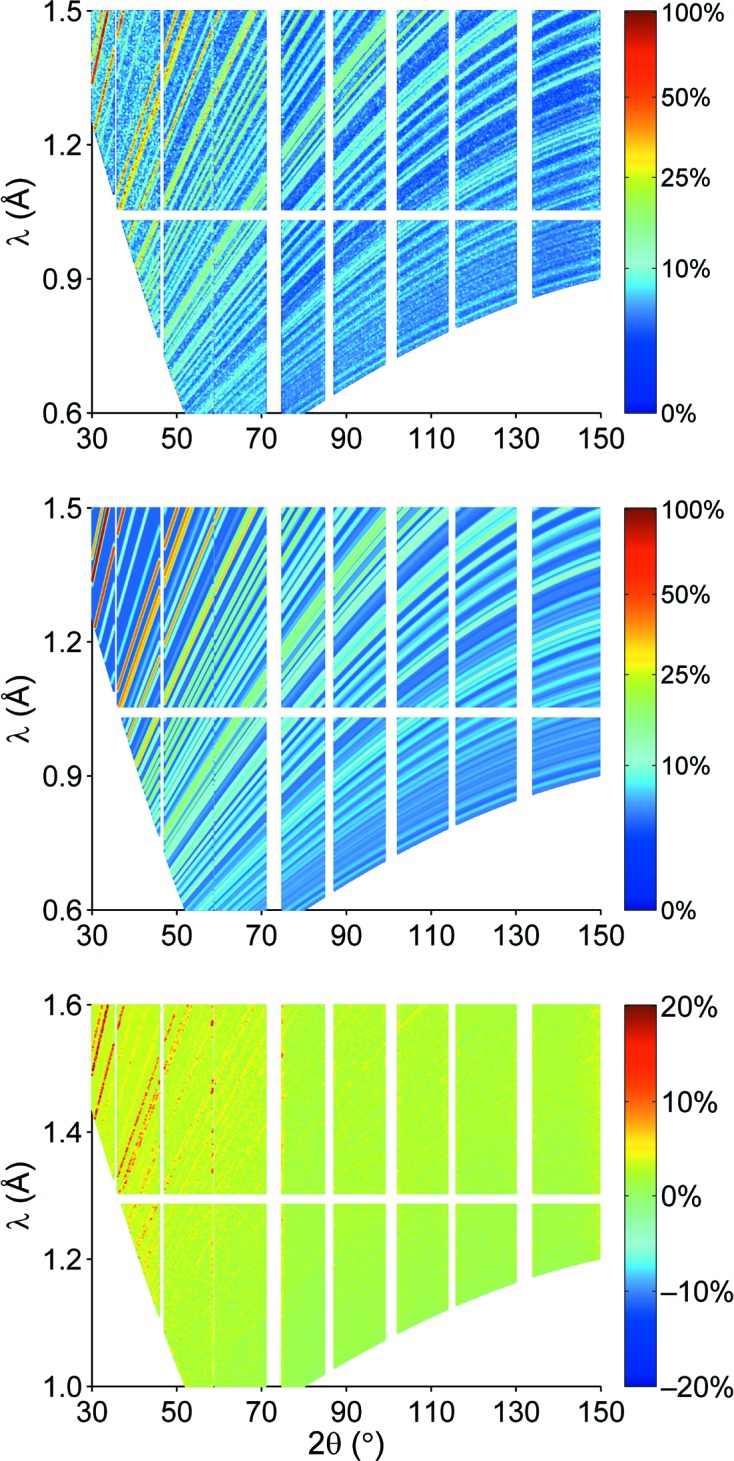
Measured (top), fitted (middle) and differential (bottom) diffraction pattern of CuNCN data from POWGEN. The color bar of each picture denotes the intensity as a percentage of the largest intensity peak in the simulated diffraction pattern.

The FWHM of each reflection has thus been handled according to the notation used in *FullProf* (http://www.ill.eu/sites/fullprof/) leading to
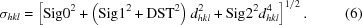
Sig0, Sig1 and Sig2 are refinable parameters defining the half-width of the peak at value *d*_*hkl*_ and DST defines an additional contribution by microstrain. The measured diffraction data and the results from refinement are shown in Fig. 8 ▸. Compared to conventional one-dimensional diffraction patterns, the statistical variation is of course more apparent in the two-dimensional distribution of the data which were measured in 7.5 h. The two-dimensional refinement yields an excellent data description as seen from the difference map in Fig. 8 ▸. The refinement parameters are given in Table 4 ▸ and indicate excellent agreement with the parameters and accuracies obtained by the standard Rietveld (*FullProf*) refinement; as expected, however, the residual value for the two-dimensional refinement is larger, thereby reflecting the much lower statistical significance per data pixel. The comparison also reveals that the conventional analysis should be typically fine for the analysis of POWGEN data which goes back to the instrument optimization with respect to resolution properties. The novel approach, however, additionally offers a more thorough check of the data quality of TOF diffractometers at current spallation sources. For an instrument like POWTEX and, likewise, for future powder diffractometers at the ESS (European Spallation Source) which will also use pulse-shaping choppers, the new approach will fully exploit the best resolution properties which would be lost by averaging for today’s standard refinement procedures.

**Figure 9:**
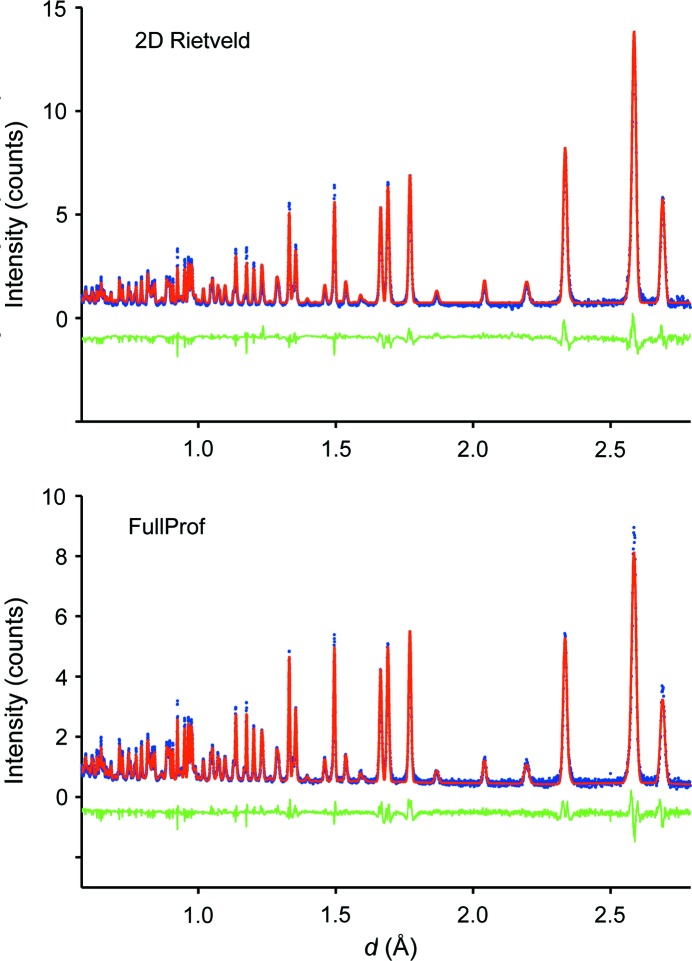
Top: conventional one-dimensional diffraction pattern of CuNCN derived from the two-dimensional Rietveld refinement. Bottom: comparison with the standard Rietveld refinement using *FullProf*.

Since the POWTEX instrument reflects, by its very design, a user-driven approach of the solid-state chemistry community to structural characterization, the developers ought to carefully react to gathered user feedback. One main concern of today’s users might be foreseeable in the unfamiliar two-dimensional diffraction pattern (Fig. 1 ▸) and especially the data comparison within the novel Rietveld strategy. We therefore point out that it is quite easy to generate all sorts of reduced plots, such as the traditional ‘Rietveld pattern’ of intensity *versus* diffraction angle, irrespective of the fact that the data treatment and refinement will be done with the full two-dimensional data for reasons of superior refinement quality. A comparison with a standard one-dimensional *FullProf* refinement (Jacobs *et al.*, 2013 ▸) is shown in Fig. 9 ▸. Furthermore, the integration of POWTEX’s intensities to a one-dimensional pattern is also possible. In combination with further developed techniques, *e.g.* nonlinear multi-bank approaches comparable to POLARIS or GEM (Hannon, 2005 ▸; Hull *et al.*, 1992 ▸), one may at least partially overcome some of the above-mentioned issues and, as a benefit, adopt the data treatment more easily for POWTEX in contemporary refinement software. However, this is out of the scope of this article and was not elaborately tested.

## 5. Conclusion and outlook   

We have demonstrated a simultaneous Rietveld refinement of angular- and wavelength-dispersive two-dimensional data sets. The latter were based on simulated Monte Carlo data using the layout of the POWTEX instrument which has been particularly optimized to benefit from a smooth but varying resolution with the highest resolution at large 2θ.

In a first test we used simulated data obtained from the *VITESS* program package based on an idealized instrument layout and the structural models of Rh_0.81_Fe_3.19_N and CuNCN as idealized samples. Similar to procedures on existing instruments, these ‘standard’ samples serve as input to determine the instrumental profile function as a function of 2θ and λ. Once established on the basis of real data, this instrumental profile function will be provided to the user. If deviations of the observed peak shape from the instrumental profile function should occur, these can then be attributed to sample effects and may be addressed accordingly.

In contrast to the POWTEX design, the TOF powder diffractometer POWGEN at the SNS in Oak Ridge, for example, has a deliberately chosen detector design which tries to minimize 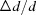 by best matching the angular contribution Δθcotθ to the relative time resolution 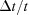. The latter is fairly independent of the wavelength owing to the moderators’ natural moderation time at short-pulse spallation sources while, as in our case, pulse shaping results in a constant absolute time resolution Δ*t*. Because POWGEN has been designed in such a way that the resolution in time and angle are matched to each other, one may expect that it is possible to reduce their measured two-dimensional data to a one-dimensional data set without severe compromises in quality. However, it is also straightforward to test the two-dimensional approach with experimental POWGEN data. For this second test, we used such an unreduced data set of a CuNCN sample measured at POWGEN and showed how to successfully apply our two-dimensional approach using these data; thus, the feasibility of the two-dimensional refinement method for experimental data has been validated. Experienced TOF diffraction users, who know that, sometimes, measured data are deliberately discarded to avoid accuracy loss introduced by data integration, might also appreciate the chance to check the data quality. Furthermore, we believe that the novel approach will have an impact not only on data analysis, since it allows more freedom to drive the instrumental design towards less complex detector arrangements and, most favorably, to the cylindrical POWTEX geometry with its axis along the beam direction. Therefore, the proposed approach seems to be of interest for instruments based on a similar geometry concept such as POLARIS at ISIS, Super-HRPD at JPARC and DREAM, which is designed for the ESS.

The distinct advantage of two-dimensional refinements with better control and analysis of the background makes a particularly interesting case for the parasitic incoherent scattering of hydrogen, which is typically inelastic and depends on the incident wavelength (Henry *et al.*, 2009 ▸; Wilson *et al.*, 2014 ▸). Such an effect should be rather obvious in two-dimensional data, in contrast to integrated one-dimensional data, and it should also be possible to model (for example, subtract) the phenomenon because of its known wavelength dependence.

With the fundamentals of two-dimensional data profiling laid out, future developments will aim at incorporating more sample effects (*e.g.* preferred orientation, absorption and such like). The given proof-of-concept will hopefully motivate the incorporation of this novel approach into existing programs such as *FullProf*, *GSAS*, *MAUD*, *TOPAS* or *JANA*, which will definitely be needed for future user applications.

## Figures and Tables

**Table 1:** Instrumental parameters to generate data sets using the Monte Carlo instrument simulation package *VITESS*

No. starting trajectories	1.5 × 10^12^
Length of neutron guide No. 1 (m)	27.078
Length of neutron guide No. 2 (m)	11.096
Double-disc pulse chopper	Two counter-rotating discs with 11/10 apertures and 75 cm diameter
Total length chopper to sample (m)	12.128
Sample	Spherical, radius = 1 cm
Detector	Cylindrical, *l* = 1.6 m, *r* = 0.8 m

**Table 2:** Crystallographic data of Rh_0.81_Fe_3.19_N and CuNCN

	Rh_0.81_Fe_3.19_N (Houben *et al.*, 2009 ▸)	CuNCN (Jacobs *et al.*, 2013 ▸)
Lattice parameters (Å)	*a* = 3.83366 (2)	*a* = 2.98908 (8), *b* = 6.1420 (3), *c* = 9.4009 (4)
Space group	*Pm* 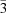 *m* (No. 212)	*Cmcm* (No. 63)
Formula units	*Z* = 1	*Z* = 4
Atomic sites	Rh (1*a*) 0 | 0 | 0	Cu (4*a*) 0 | 0 | 0
	N (1*b*) ½ | ½ | ½	C (4*c*) 0 | 0.3889 (8) | ¼
	Fe (3*c*) 0 | ½ | ½	N (8*f*) 0 | 0.3826 (4) | 0.3815 (3)
Temperature	*T* = 300 K	*T* = 17 K

**Table 3:** Results of the pattern fitting for the simulated data of Rh_0.81_Fe_3.19_N and CuNCN

	Rh_0.81_Fe_3.19_N	CuNCN
No. parameters	3	5
No. data points	2.16 million	2.16 million
No. reflections	68	389
Calculation time (min)†	< 2	≃ 30
Scale	2.435 (1) × 10^−3^	2.664 (1) × 10^−4^
Background	1.79 (7) × 10^−4^	1.96 (2) × 10^−4^
Lattice parameters (Å)	*a* = 3.83364 (1)	*a* = 2.98905 (1), *b* = 6.14192 (2), *c* = 9.40087 (2)
*R*_p_	0.060	0.050

† ASUS K73S Notebook with Intel Core i5-2410M (2 Cores @ 2.3 GHz) and 6 GB of RAM.

**Table 4:** Results of the two-dimensional pattern fitting for the experimental data of CuNCN (POWGEN) in comparison to the *FullProf* refinement

	CuNCN (two-dimensional Rietveld)	CuNCN (focused)
No. parameters	5	27
No. data points	0.62 million	5920
No. reflections	515	276
Calculation time (min)†	60	≃ 1
Scale	0.0160 (1)	1.021 (6)
Background	Single value of 0.840 (3)	Interpolation between 30 selected points
Lattice parameters (Å)	*a* = 2.98920 (7), *b* = 6.1423 (2), *c* = 9.4012 (3)	*a* = 2.98908 (8), *b* = 6.1420 (3), *c* = 9.4009 (4)
*R*_p_	0.25	0.05

† ASUS K73S Notebook with Intel Core i5-2410M (2 Cores @ 2.3 GHz) and 6 GB of RAM.
